# Kinetics, Moderators and Reference Limits of Exercise-Induced Elevation of Cardiac Troponin T in Athletes: A Systematic Review and Meta-Analysis

**DOI:** 10.3389/fphys.2021.651851

**Published:** 2021-03-26

**Authors:** Feifei Li, Will G. Hopkins, Xuejing Wang, Julien S. Baker, Jinlei Nie, Junqiang Qiu, Binh Quach, Kun Wang, Longyan Yi

**Affiliations:** ^1^Department of Sport, Physical Education and Health, Hong Kong Baptist University, Hong Kong, China; ^2^Centre for Health and Exercise Science Research, Hong Kong Baptist University, Hong Kong, China; ^3^Institute for Health and Sport, Victoria University, Melbourne, VIC, Australia; ^4^Clinical Laboratory, Civil Aviation General Hospital, Beijing, China; ^5^School of Health Sciences and Sports, Macao Polytechnic Institute, Macao, China; ^6^College of Sport Science School, Beijing Sport University, Beijing, China; ^7^College of Physical Education, Hebei Normal University, Shijiazhuang, China; ^8^China Institute of Sport and Health Science, Beijing Sport University, Beijing, China

**Keywords:** cardiac biomarker, reference range, running, cycling, swimming, triathlon, time-course, marathon

## Abstract

**Background:** Kinetics, moderators and reference limits for exercise-induced cardiac troponin T (cTnT) elevations are still unclear.

**Methods:** A systematic review of published literature was conducted adhering to the Preferred Reporting Items for Systematic Review and Meta-Analyses (PRISMA) guidelines. Studies reporting high-sensitivity cardiac troponin T (hs-cTnT) concentrations before and after a bout of exercise in athletes were included and analyzed. The final dataset consisted of 62 estimates from 16 bouts in 13 studies of 5–1,002 athletes (1,421 in total). Meta-analysis was performed using general linear mixed modeling and Bayesian inferences about effect magnitudes. Modifying fixed-effect moderators of gender, age, baseline level, exercise duration, intensity and modalities were investigated. Simulation was used to derive 99th percentile with 95% limits of upper reference ranges for hs-cTnT of athletic populations.

**Results:** The mean and upper reference limits of hs-cTnT before exercise were 4.4 and 19 ng.L^−1^. Clear increases in hs-cTnT ranging from large to very large (factor changes of 2.1–7.5, 90% compatibility limits, ×/÷1.3) were evident from 0.7 through 25 h, peaking at 2.9 h after the midpoint of a 2.5-h bout of running, when the mean and upper reference limit for hs-cTnT were 33 and 390 ng L^−1^. A four-fold increase in exercise duration produced a large clear increase (2.4, ×/÷1.7) in post-exercise hs-cTnT. Rowing exercise demonstrated an extremely large clear reduction (0.1 ×/÷2.4).

**Conclusions:** The kinetics of cTnT elevation following exercise, the positive effect of exercise duration, the impact of exercise modality and 99th upper reference limits for athletic populations were reasonably well defined by this meta-analysis.

## Introduction

It has been well established that regular physical activity is beneficial for cardiovascular health, but there are concerns that exercise can also be too strenuous and detrimental to health (Parry-Williams and Sharma, 2020). Some of the concerns arise from the growing evidence of cardiac-specific biomarker elevations after acute bouts of exercise (Le Goff et al., 2020). Cardiac troponin (cTn), including isoforms of cardiac troponin T (cTnT), and I (cTnI), is released only from the cardiomyocytes (Collinson et al., 2001). As a specific biomarker of myocardial injury, serum concentration of cTn above the 99th percentile upper reference limit for a healthy population is key, in conjunction with other clinical elements, to establish the diagnosis of acute myocardial infarction (Thygesen et al., 2018). Exercise-induced elevations of cTn have been observed with some concentrations above the upper reference limit in “apparently healthy” athletes using earlier generations of the assay (Shave et al., 2010). Because of this observation, it has been debated if a physiological or pathological process was responsible for the elevations observed. The absence of related evidence, especially the lack of reference ranges for baseline and post-exercise levels of cTn in athletic populations, has made meaningful conclusions difficult. Along with the introduction and application of high-sensitivity assays with much lower detectable concentration capability, a higher proportion of athletes were detected or tested positive for elevations in high-sensitivity cardiac troponin (hs-cTn) prior to and following exercise (Gresslien and Agewall, 2016). Since hs-cTn has been interpreted as a quantitative assessment for cardiomyocyte damage (Collet et al., 2020), with even low-level elevations in healthy populations used to predict further cardiovascular risks associated with chronic myocardial injury (Seliger et al., 2017; Aakre et al., 2020), it is necessary to reconsider our understanding of the underlying mechanisms and clinical implications related to hs-cTn elevations following exercise in athletes.

The kinetics of exercise-induced cTn elevation has been investigated by researchers for evidence to differentiate between the pathological and physiological release processes. In clinical settings, cTn shows a consistent pattern of a rapid rise and a slow decline following myocardial infarction (Thygesen et al., 2018). The kinetics of exercise-induced cTn changes depend on exercise duration (Baker et al., 2019). Published cardiac troponin release profiles have been limited to single exercise bouts with multiple exercise durations but few time points and usually small sample sizes. Therefore a meta-analysis is required to overcome these limitations (Tian et al., 2012; Baker et al., 2019). In previous meta-analyses, the post-exercise cTn data were pooled according to the positive concentration rates above the identified detection limit (Shave et al., 2007; Donaldson et al., 2019). This was a semi-qualitative method that may not be sufficiently robust for the new assay. Therefore, the release kinetics and factors affecting hs-cTn following exercise need further investigation.

In order to understand the underlying mechanisms and related clinical implications, several studies have identified potential moderators of exercise-induced cTn elevation. Overall, hs-cTn concentrations was impacted: by exercise duration (Kleiven et al., 2019), intensity (Richardson et al., 2018; Martínez-Navarro et al., 2020) and workload (Cirer-Sastre et al., 2019); maturational status (Martínez-Navarro et al., 2020; Cirer-Sastre et al., 2021); training experience (Tian et al., 2012); gender (Kong et al., 2017; Legaz-Arrese et al., 2017); basal level of cTn (Legaz-Arrese et al., 2015a,c); as well as environment (Li et al., 2016). The possibility of interaction of the moderators needs further investigation. There are growing numbers of assays for the determination of hs-cTn, most of which are for the I variant. However, there is only one high-sensitivity assay for T, allowing for standardization across studies (Omland and Aakre, 2019). Therefore, the purpose of this study was to conduct a meta-analysis to investigate the kinetics, potential moderators and reference limits of exercise-induced cTnT elevations in athletic populations.

## Methods

### Literature Search

A literature search of peer-reviewed, English-language papers published in the Web of science, PubMed and MEDLINE between January 2010 and December 2019 was conducted. The key words used were “high-sensitivity cardiac troponin T,” or “cardiac troponin T,” or “cardiac troponin,” combined with “exercise,” or “acute exercise,” or “endurance.” The initial screening and selection was untaken by two authors (KW and FL) according to the presented inclusion and exclusion criteria. Following initial selection, potentially eligible studies were further identified by screening the titles and abstracts. If there was a dispute between the two authors, the paper was sent to a third author (YL) for a final decision. The reference list of the included studies or the paper citing included studies was also reviewed for suitable references that did not appear in the initial database search.

### Inclusion and Exclusion Criteria

1. The setting was acute exercise, either field- or laboratory-based, using athletes (elite, professional, or recreational), with adequate description of exercise mode (running or marathon, cycling, swimming, triathlon, and rowing), exercise duration (in min or h), exercise mean heart rate (in min^−1^, continuously recorded during exercise), and athletic status (age, gender, weight, height, VO_2max_, training experiences and training volume per day or per week) of participants. Studies of cardiac-stress or a VO_2max_ test with exercise <30 min were excluded.
2. The participants were able to describe personal history of cardiovascular disease and showed a normal electrocardiogram, or echocardiogram at rest conducted by professionals.
3. A blood test for the determination of high-sensitivity cTnT (hs-cTnT) was used. Concentrations (mean and standard deviation or median and quartiles) of hs-cTnT pre-exercise and for at least one time point post-exercise were reported.

### Quality Assessment

The quality of potentially selected studies were assessed by Quality Assessment Tool for Before-After (Pre-Post) Studies With No Control Group of 12 binary items (National Heart Lung and Blood Institute, 2014). The assessment was conducted by two authors (KW and FL) and discrepancy was discussed with a third author (YL) for resolve.

### Data Extraction

Given the fact that the SD of the concentrations of hs-cTnT sometimes exceeded the mean, we assumed that the means and SDs of log-transformed concentrations would be approximately normally distributed and would therefore provide more trustworthy meta-analyzed estimates (after back-transformation) than the raw means and SDs. The raw mean and SD for each concentration of hs-cTnT were therefore converted to back-transformed means and factor SD of log-transformed concentrations. The assumption of normality of log-transformed concentrations allowed estimation of the 100 percentiles of concentration for given log-transformed values of means and factor SD; the mean and SDs of the back-transformed percentiles were then compared with the raw mean and SDs using the Solver in a Microsoft Excel spreadsheet to find the values of log-transformed mean and factor SDs that gave the observed raw mean and SD. In studies where the concentrations of hs-cTnT were reported as median and quartiles, the log-transformed median and log-transformed quartiles provided respectively estimates of the log-transformed means and log-transformed factor SD, the latter by dividing half the interquartile range by the appropriate t statistic; in some estimates with low medians (four pre-exercise, one at 72 h post-exercise), the lower quartile was the same or a little less than the median (indicating a substantial proportion of concentrations at the threshold of detection), so the SD was derived via the difference between median and upper quartile.

### Meta-Analytic Model

We used an approach to the modeling similar to that of a recent meta-analysis (Patten et al., 2020) as outlined previously (Hopkins et al., 2009). The Statistical Analysis System (University Edition of SAS Studio, Version 9.4, SAS Institute, Cary, NC) was used to perform the analysis with the general linear mixed-model procedure (Proc Mixed). Separate meta-analyses were performed for the study-estimates of mean and SDs of hs-cTnT; the dependent variable was the log of the mean and the log of the log of the factor SD, respectively, but the modifying fixed effects and the random effects were otherwise the same for both analyses. Several fixed-effect moderators were investigated, but the most parsimonious had the following fixed effects: a nominal effect for time point (eight levels: pre-exercise, and ≤1, >1, >2, >4, >10, >20, >40 h for adjusted post-exercise time, which was the reported post-exercise time plus half the exercise duration); a linear numeric variable for gender (the proportion of males in the study) for pre-exercise values and another for post-exercise values; a linear numeric effect of age, again with a separate effect for pre- and post-exercise values; linear numeric effects of pre-exercise log-transformed hs-cTnT, mean exercise heart rate, and log-transformed exercise duration; and a nominal effect for exercise modality (five levels: cycling, rowing, running, swimming, and triathlon). The random effects were: the identity of the pre-exercise bout (identifying each study's pre-exercise values, with a different value for each bout for two studies where the subjects performed two bouts (Li et al., 2020) or three bouts (Legaz-Arrese et al., 2015b); the identity of the post-exercise bout (again with a different value for a second or third bout); and the identity of each post-exercise time point within each bout (46 levels, with each study contributing 1–6 levels). Each study estimate was weighted by the inverse of the square of its standard error, and the random effects were estimated by setting the residual variance to unity (Yang, 2003). The standard error for the meta-analysis of the log of mean concentration was the standard error of the mean [the log of the factor SD divided by  (sample size)]; the standard error for the meta-analysis of the log of the log of the factor SD was 1/ (2(degrees of freedom – 1)), a formula verified with a simulation spreadsheet.

#### Outcome Statistics

Meta-analyzed mean and SDs of pre-exercise concentrations were predicted for males of age 37.5 year (the approximate mean across the studies); post-exercise means and SDs were predicted for males of age 37.5 year running for 2.5 h with a mean heart rate of 155 min^−1^ and a pre-exercise hs-cTnT of 4.4 ng L^−1^ (the approximate means across the studies). Meta-analyzed means and SDs were back-transformed to actual concentrations and factor SD, and modifying effects were back-transformed to factor effects. The modifying effects of age were expressed for a 25-year difference (approximately two between-subject SD averaged across studies); the modifying effect of pre-exercise hs-cTnT concentration was estimated for a 3.2-fold difference in concentration (approximately two between-subject SDs, averaged across studies *via* log-transformation and variances); modifying effects of exercise were estimated for a four-fold difference in duration and a difference in mean heart rate of 20 min^−1^ (both approximately 2 SD of the study means). Each modality of exercise was compared with running. Between- and within-bout SD derived from the random effects were combined (*via* variances) to give an SD representing differences between study settings (different studies and time points), except that the within-bout SD in the meta-analysis of SDs was zero (a factor SD of 1).

In the absence of clinically relevant values for an exercise-associated increase in hs-cTnT, magnitudes of meta-analyzed modifying effects were evaluated via standardization using magnitude thresholds provided by the appropriate pre-exercise between-subject SDs (Hopkins et al., 2009), here the square root of the mean of the squared log of the factor SD. The thresholds for small, moderate, large, very large and extremely large increases were 0.2, 0.6, 1.2, 2.0, and 4.0 times this SD (Hopkins et al., 2009); in factor units, the mean SD was 1.62, the thresholds for increases were 1.12, 1.41, 2.0, 3.2, and 10, and their inverses provided thresholds for reductions of 0.89, 0.71, 0.50, 0.32, and 0.10. Square roots of these thresholds (half their log-transformed values) were used for evaluation of the magnitude of the random-effect SDs in the meta-analysis of mean hs-cTnT and for evaluation of the magnitude of the meta-analyzed SDs (Smith and Hopkins, 2011). Assessment of magnitudes of modifying effects in the meta-analysis of SDs was performed for the effects expressed as ratios of the log-transformed factor SD, since the meta-analysis was performed via the log-transformed log of the factor SD. Standardization was used to derive the magnitude thresholds for the ratios by regarding the SD in the denominator of the ratio as the standardizing SD. If an effect was responsible for additional variance, d^2^, this variance would add to the denominator variance D^2^ to give the numerator variance, D^2^+d^2^. The ratio of the SD would therefore be $\sqrt{\;}\left(\left({\text{D}}^{2}+{\text{d}}^{2}\right)/{\text{D}}^{2}\right)=\sqrt{\;}\left(1+{\text{d}}^{2}/{\text{D}}^{2}\right)$. For the magnitude thresholds defined by standardization for SDs, d has values D times half the usual thresholds of 0.2, 0.6, 1.2, 2.0, and 4.0; the resulting threshold ratios for an increase in SD are 1.005, 1.04, 1.17, 1.41, and 2.2, and their inverses for a decrease in SD are 0.995, 0.96, 0.86, 0.71, and 0.45.

Uncertainty in the estimates of effects is presented as 90% compatibility limits. Probabilistic decisions about true (large-sample) magnitudes accounting for the uncertainty were based on one-sided hypothesis tests of substantial magnitudes (Lakens et al., 2017). The *p* value for rejecting a hypothesis of a given magnitude was the area of the sampling t distribution of the effect statistic with values of that magnitude. Bayesian analysis with a minimally informative prior (Hopkins, 2019). The *p* value is reported qualitatively using the following scale: ≥ 0.25, possibly; ≥ 0.75, likely; ≥ 0.95, very likely; ≥ 0.995, most likely (Hopkins et al., 2009). This scale was also used to interpret the posterior probability of a true trivial effect, which is given by the area of the sampling distribution in trivial values. If neither hypothesis was rejected, the magnitude of the effect was considered to be unclear, and the magnitude of the effect is shown without a probabilistic qualifier.

#### Publication Bias and Outliers

The solutions for the random effects were combined and expressed as t statistics for plotting against the standard error of their corresponding study-estimate (Hopkins et al., 2009). By accounting for uncertainty in the estimates and for the contribution of study covariates, this plot is an improvement over the usual funnel plot. In the meta-analysis of hs-cTnT means, there was no noticeable asymmetry in the scatterplot of the pre-exercise random effect for bout, but there was slight asymmetry in the post-exercise random effect for bout, consistent with the possibility of larger values of mean hs-cTnT in studies with smaller sample sizes. All *t* values were between ± 2.0, well below the outlier threshold of 3.5 (Hopkins et al., 2009). In the meta-analysis of hs-cTnT SDs, scatterplots showed the possibility of larger SDs in pre- and post-exercise bouts of studies with smaller sample sizes. T values were all between ±3.0.

#### Reference Limits for hs-cTnT

Simulation was used to derive upper limits for reference ranges for hs-cTnT from the meta-analyzed means and SDs. Assumption of normality of the sampling distribution of the setting variance in both meta-analyses allowed a point-estimate value of the log-transformed mean and log-transformed factor SD to be drawn for a randomly chosen setting using randomly drawn normal deviates. Assumption of normality of log-transformed hs-cTnT then allowed a single value of hs-cTnT to be drawn for this setting. Another value was drawn, but this time, randomly chosen values determined by the sampling standard errors of all the contributing means and SDs were included. A total of 400,000 samples were thereby drawn, and the 99th percentile of the values with and without inclusion of the standard errors were derived. With the further assumption that the 99th percentile was normally distributed, its standard error was derived from the two 99th percentiles and used to determine the compatibility limits of the point-estimate 99th percentile with 95% limits. This process was performed for predicted values of male athletes of age 25, 37.5, and 50 years (approximately one SD each side of the mean age) for pre-exercise hs-cTnT; for post-exercise hs-cTnT the process was repeated for these three ages for each of three durations of running of 1.25, 2.5, and 5.0 h (approximately one factor SD each side of the log mean of bout duration) at the bout-mean mean heart rate of 155 min^−1^ with a pre-exercise bout-mean mean concentration of 4.4 ng.L^−1^.

## Results

**Figure 1:**
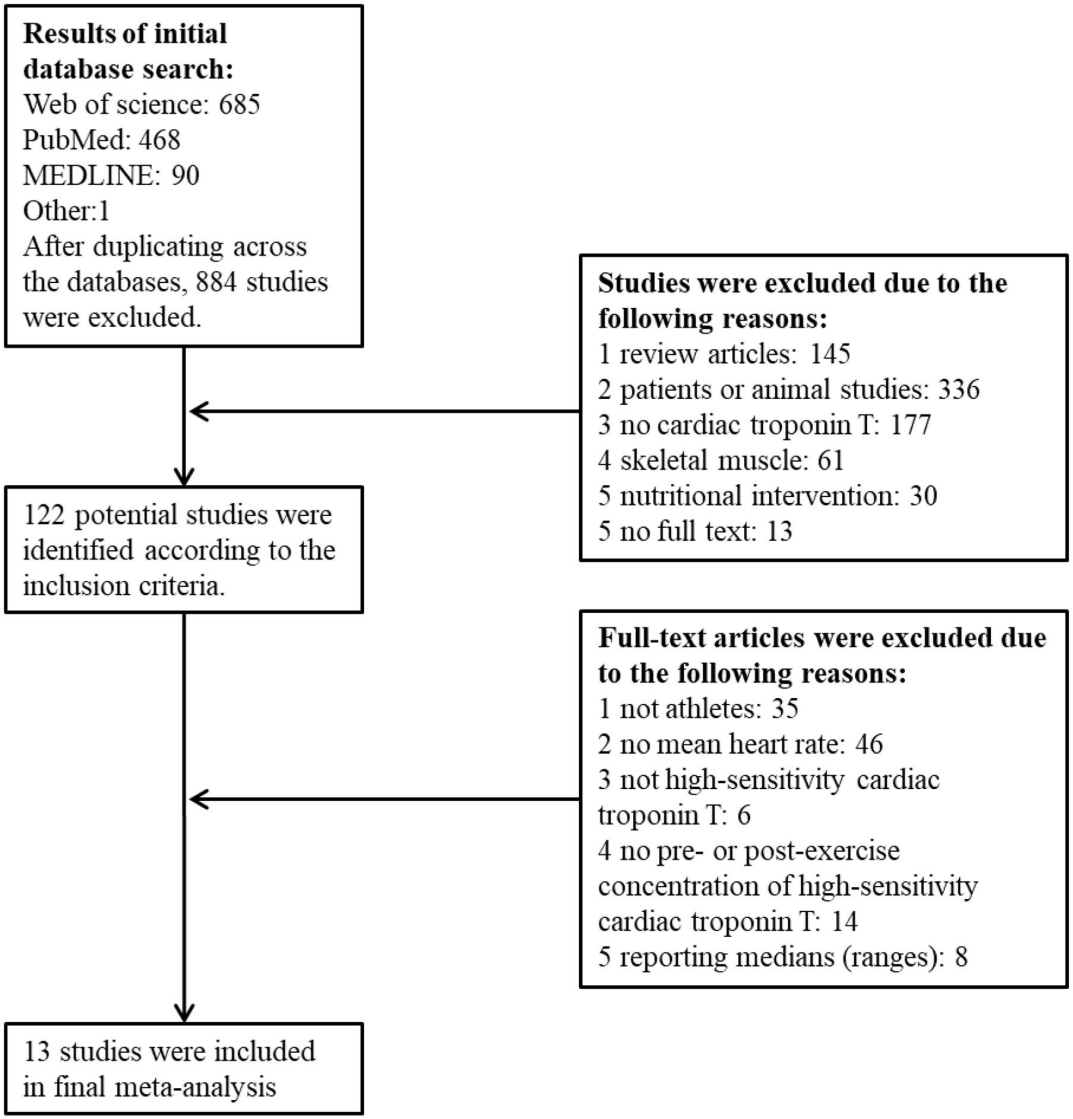
PRISMA flow chart outlining literature search, inclusion and exclusion of studies.

The search process is outlined in Figure 1. Thirteen studies were included with 16 bouts of exercise to the final dataset consisted of 62 estimates of hs-cTnT comprising of 5–1,002 participants, 1,421 in total. Descriptive statistics for these studies are outlined in Table 1. All studies targeted athletic populations, characterized by training experience (5.4–7 years) (Legaz-Arrese et al., 2015b; Stewart et al., 2015; Li et al., 2017, 2020), competitions history (7 races per 5 yeas) (Kleiven et al., 2019), or training volume per week by hours (2.4–13 h) (Aagaard et al., 2014; Legaz-Arrese et al., 2015b; Stewart et al., 2015; Aakre et al., 2018; Tahir et al., 2020) or by distance (32.5–54.1 km) (Scherr et al., 2011; Grabs et al., 2015; Li et al., 2017, 2020; Richardson et al., 2018). Two studies did not mention training status information listed above, but used cyclists with VO_2max_ of 64.8 ± 4.1 ml kg min^−1^ (Stewart et al., 2014) and competitors in a professional rowing race (Frias et al., 2018). All authors described the health status of participants with no cardiovascular disease history or no medication by self-report (Stewart et al., 2014, 2015; Tahir et al., 2020), or medical screening by electrocardiogram or echocardiogram at rest by professionals (Scherr et al., 2011; Aagaard et al., 2014; Grabs et al., 2015; Legaz-Arrese et al., 2015b; Li et al., 2017, 2020; Aakre et al., 2018; Kleiven et al., 2019). Two studies did not mention health status information listed above, but competitors of Brighton Marathon (Richardson et al., 2018) and professional rowing race (Frias et al., 2018). The number of study-estimates in each time group (pre-exercise and seven post-exercise) were 16, 7, 8, 7, 9, 3, 9, and 3.

**Table 1:** Descriptive statistics of studies included in the meta-analysis.

**References**	**Sample size**	**Proportion of males**	**Mean age (y)**	**Mean weight (kg)**	**Exercise modality**	**Exercise duration (h)**	**Exercise heart rate (min^**−1**^)**	**Pre-exercise hs-cTnT** **(ng.L**^****−1****^**)**		**Time points of sampling post-exercise (h)**
								**Original^a^**	**Via log-trans.^b^**	
Tahir et al. (2020)	30	1.00	45	79	Triathlon	3.3	155^c^	7.0 ± 4.0	6.4 ×/÷ 1.5	2
Richardson et al. (2018)	52	0.75	39	76	Running	4.4	160	5.6 ± 3.3	4.8 ×/÷ 1.7	0
Li et al. (2017)	21	0.95	23	64	Running	1.5	158	4.7 ± 1.9	4.4 ×/÷ 1.5	0, 4, 24
Stewart et al. (2015)	15	1.00	28		Cycling	1.0	168	6.0 ± 0.6	5.6 ×/÷ 1.5	0.5
Legaz-Arrese et al. (2015b)	15	1.00	35	71	Swimming	1.0	150	4.2 ± 1.3	4.0 ×/÷ 1.3	0, 1, 3, 6, 12, 24
Legaz-Arrese et al. (2015b)	15	1.00	35	71	Cycling	1.0	157	4.3 ± 1.3	4.1 ×/÷ 1.3	0, 1, 3, 6, 12, 24
Legaz-Arrese et al. (2015b)	15	1.00	35	71	Running	1.0	164	4.1 ± 1.5	3.9 ×/÷ 1.4	0, 1, 3, 6, 12, 24
Stewart et al. (2014)	8	1.00	23	73	Cycling	2.0	168	5.2 ± 0.7	4.9 ×/÷ 1.5	1
Li et al. (2020)	12	0.92	24	63	Running	1.5	160	8.3 ± 12.6	4.6 ×/÷ 3.0	0, 1, 4, 24, 48
Li et al. (2020)	12	0.92	23.5	63	Running	1.5	162	6.9 ± 8.1	4.5 ×/÷ 2.5	0, 1, 4, 24, 48
Kleiven et al. (2019)	1002	0.78	47	82	Cycling	3.7	157	3.0 ± 0.9	3.0 ×/÷ 1.7	3, 24
Frias et al. (2018)	5	0.80	44	85	Rowing	16.4	120	3.0 ± 0.3	3.0 ×/÷ 1.2	0
Aakre et al. (2018)	97	0.76	43	83	Cycling	4.2	158	4.1 ± 2.3	4.1 ×/÷ 1.6	0, 3, 24
Grabs et al. (2015)	20	1.00	45		Running	4.0	150	6.0 ± 3.0	6.0 ×/÷ 1.5	0
Aagaard et al. (2014)	42	1.00	51	82	Running	3.3^d^	127	3.2 ± 1.9	3.2 ×/÷ 2.3	0.5
Scherr et al. (2011)	102	1.00	42		Running	3.8	156	3.3 ± 1.4	3.3 ×/÷ 1.8	0, 24, 72

*hs-cTnT, serum concentration of high-sensitivity cardiac troponin T*.

a *Data are mean ± standard deviation (SD) (Studies 1–7) or median ± estimated SD (Studies 8–13)*.

b *Data are back-transformed mean ×/÷ factor SD estimated from normally-distributed log-transformed values*.

c *Missing value imputed as the mean of other studies*.

d *Missing value estimated from similar studies*.

### Mean and SD of hs-cTnT

**Figure 2:**
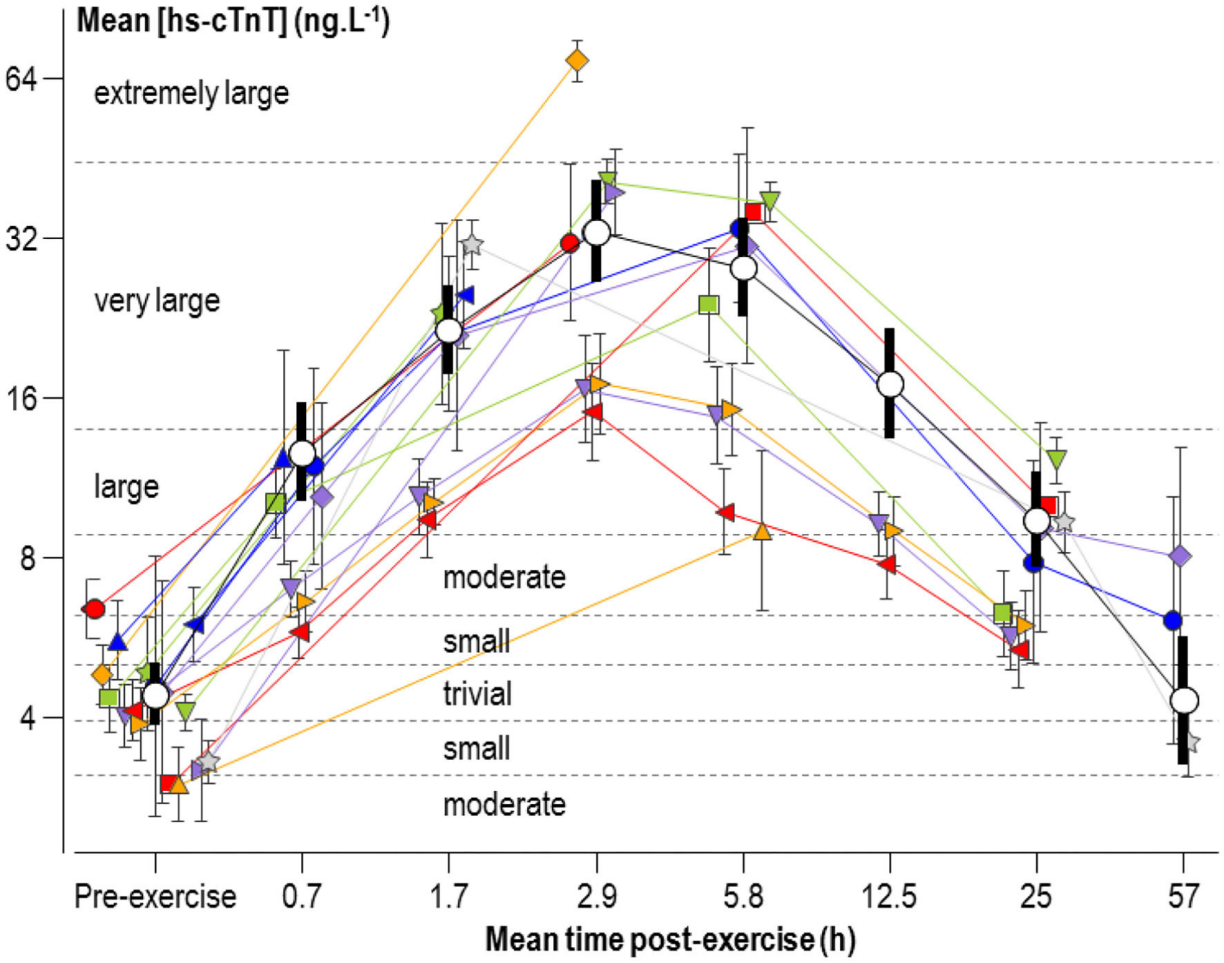
Individual study-estimate and meta-analyzed predicted mean serum concentrations of high-sensitivity cardiac troponin T (hs-cTnT), plotted against the mean adjusted post-exercise time (post-exercise time plus half the duration of exercise). The meta-analyzed pre-exercise mean is for the average male athlete. The meta-analyzed post-exercise means are for the average male running for 2.5 h with a mean heart rate of 155 min^−1^ and a pre-exercise hs-cTnT of 4.4 ng L^−1^. Bars are 90% compatibility intervals.

The mean concentration of hs-cTnT pre- and post-exercise in individual studies and the meta-analyzed predicted mean are shown in Figure 2, plotted against the mean value of each post-exercise time group. Changes from the pre-exercise mean for all but the last time point ranged from large to very large increases and most likely substantial (Table 2). The change at the last time point was trivial but unclear (90% compatibility limits ranging from a small reduction to a small increase).

**Table 2:** Meta-analyzed modifying effects on pre- and post-exercise serum concentration of high-sensitivity cardiac troponin T (hs-cTnT), and the random effects expressed as standard deviations (SD).

	**Factor effect or SD, ×/÷90%CL**	**Decision^h^**
**Modifying effects on pre-exercise hs-cTnT**		
Female/male	0.5, ×/÷3.1	Moderate ↓
Age^a^	0.9, ×/÷1.4	Small ↓
**Modifying effects on post-exercise hs-cTnT**		
Female/male	3.4, ×/÷5.6	Very large ↑
Age^a^	0.7, ×/÷1.8	Moderate ↓
Pre-exercise hs-cTnT^b^	1.5, ×/÷2.3	Moderate ↑
Exercise heart rate^c^	0.9, ×/÷1.5	Trivial
Exercise duration^d^	2.4, ×/÷1.7	Large ↑^***^
**Mode comparisons**		
Cycling/running	0.9, ×/÷1.3	Small ↓
Swimming/running	0.9, ×/÷1.7	Trivial
Triathlon/running	0.8, ×/÷1.9	Small ↓
Rowing/running	0.1, ×/÷2.4	Extremely large ↓^****^
**Post-exercise**^e^**/pre-exercise hs-cTnT**		
at 0.7 h	2.9, ×/÷1.3	Large ↑^****^
at 1.7 h	4.9, ×/÷1.3	Very large ↑^****^
at 2.9 h	7.5, ×/÷1.3	Very large ↑^****^
at 5.8 h	6.4, ×/÷1.3	Very large ↑^****^
at 12.5 h	3.8, ×/÷1.3	Large ↑^****^
at 25 h	2.1, ×/÷1.3	Large ↑^****^
at 57 h	1.0, ×/÷1.4	Trivial
**Random-effect SD**^f^		
Between bouts (pre-exercise)	1.3, ×/÷ 1.1	Moderate^***^
Between bouts (post-exercise)	1.2, ×/÷1.2	Small
Within bouts (post-exercise)	1.1, ×/÷1.1	Small^**^
Between settings (post-exercise)^g^	1.2, ×/÷1.2	Moderate

*90%CL, 90% compatibility limits; ↑, increase; ↓, decrease*.

a *Effect of a 25-year difference (~2 between-subject SD)*.

b *Effect of a 3.2-fold difference (~2 between-subject SD)*.

c *Effect of a 20-min^−1^ difference (~2 between-study SD)*.

d *Effect of a four-fold difference (~2 between-study SD)*.

e *Times shown are post-exercise time plus half the exercise duration*.

f *×/÷90%CL are approximate*.

g *Combination of the post-exercise between- and within-bout SD*.

h *Observed magnitude with magnitude-based decision*.

Clear effects are shown with the probability of a true substantial magnitude

** (likely,

*** very likely,

**** *most likely)*.

**Figure 3:**
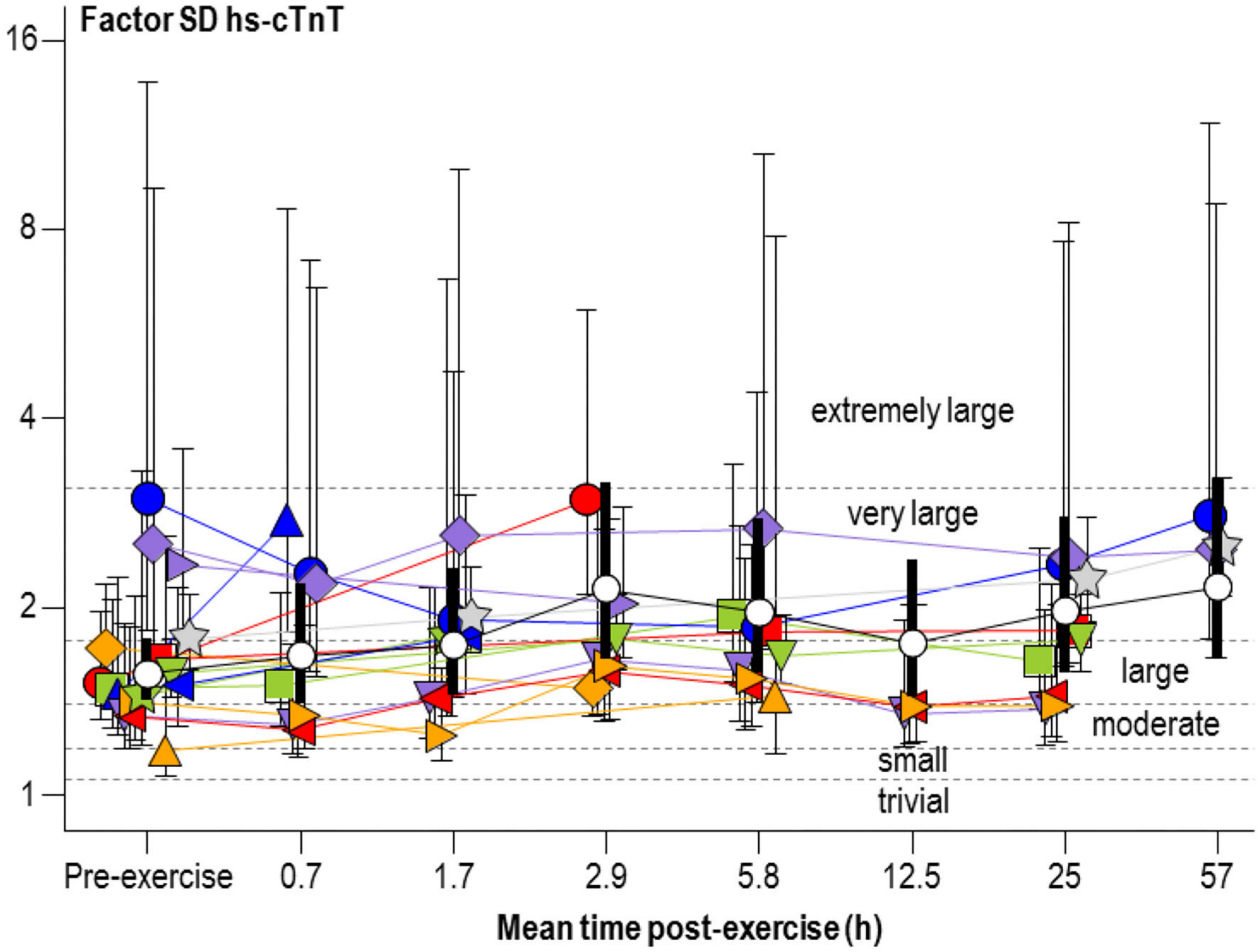
Individual study-estimate and meta-analyzed predicted factor standard deviations (SD) of serum concentration of high-sensitivity cardiac troponin T (hs-cTnT) for athletes and exercise shown in Figure 2 Bars are 90% compatibility intervals.

Figure 3 shows a plot for the factor SD of hs-cTnT similar to Figure 2. Changes from the pre-exercise SD were unclear, with the exception of the comparison of the 2.9- and 57-h time groups (both with factor changes of 1.7, 90% compatibility limits, ×/÷1.6), which represent very large increases and very likely substantial.

### Modifying and Random Effects for hs-cTnT

As shown in Table 2, studies with a higher proportion of males and older age had substantial but unclear reductions in baseline hs-cTnT. For the modifying effects on the changes in hs-cTnT post-exercise, females experienced an additional very large increase, but this effect had huge uncertainty and was unclear. Athletes with younger age and higher pre-exercise concentration of hs-cTnT experienced a moderate increase, but these effects were also unclear, as was the trivial effect of exercise heart rate. Exercise duration produced a well-defined large increase in post-exercise hs-cTnT. Compared with running, there was an extremely large clear reduction in rowing.

The random-effect SD, representing differences in concentration of hs-cTnT that were not explained by the meta-analytic predictors, showed clear substantial differences between bouts before exercise; the differences between and within bouts and their combination (between settings) were also substantial but less clear.

In the meta-analysis of factor SD, the modifying effects of gender, age, pre-exercise hs-cTnT and exercise parameters ranged from moderate to extremely large (factor changes of 0.1–1.8, 90% compatibility limits, ×/÷1.7–36). However, all these modifying effects were unclear.

### Upper Reference Limits for hs-cTnT

Although the modifying effects of gender and age on pre- and post-exercise mean hs-cTnT were unclear, it is nevertheless important to adjust for these substantial effects, when estimating reference limits. Table 3 shows the upper 99th-percentile reference limits of male runners in three age groups for pre- and post-exercise hs-cTnT. Exercise duration had a large modifying effect on post-exercise hs-cTnT, and Table 3 shows reference limits for three exercise durations.

**Table 3:** Meta-analyzed mean and upper reference limit (99th percentile) of serum concentration of high-sensitivity cardiac troponin T (hs-cTnT) in male athletes of three ages before and adjusted 2.5 h after running^a^ for three durations with a mean heart rate of 155 min^−1^.

**Age (year)**	**hs-cTnT concentration (ng.L**^****−1****^**)**	
	**Mean**	**99th percentile (95%CI)**
**Before exercise**		
25	4.8	23 (21–24)
37.5	4.4	19 (18–20)
50	4.1	17 (15–19)
**After 1.25 h of running**		
25	26	340 (250–470)
37.5	21	140 (97–190)
50	18	67 (27–160)
**After 2.5 h of running**		
25	40	1,300 (590–3,000)
37.5	33	390 (300–500)
50	27	160 (87–290)
**After 5 h of running**		
25	61	7,400 (380–140,000)
37.5	51	1,500 (610–3,800)
50	42	450 (190–1,100)

*95% CI, 95% compatibility interval*.

a *Actual times after running for 1.25, 2.5, and 5 h are 1.9, 1.3, and 0 h, respectively*.

*Post-exercise values are for pre-exercise hs-cTnT concentration of 4.4 ng.L^−1^*.

## Discussion

The meta-analysis overcomes the limitations of previous single studies using one exercise bout and a few blood sampling times, mostly with small sample sizes. In summary, there was a rapid rise and slow decline in hs-cTnT following exercise: the elevation was evident less than an hour following exercise, peaked after a few hours, and returned to normal 2–3 days later, with small changes from baseline still possible. Exercise duration was a clear moderator of exercise-induced hs-cTnT elevation. Mean concentrations were clearly lower in rowing compared with running. Increased individual variations of cTnT elevation response to exercise were evident at peak, as well as 2–3 days later without conclusive moderators. The meta-analyses of means and SDs provided upper reference limits for hs-cTnT of athletes over a range of ages at baseline and peak post running for a range of durations.

In a clinical setting, the early release of cTn from the cytosolic pool or loosely bound pool has demonstrated a consistent pattern of a rapid rise, culminating in a peak followed by a decline with a half-life of no more than 6 h, which together represent reversible injury (Thygesen et al., 2018). After the initial elevation, any release from the structural pool could obscure the decline and persist for 10–14 days, which represented irreversible myocardial injury (Mair et al., 2018). Such persistent elevations reflected continuing structural degradation (Katus et al., 1991). The new assay has improved the detection speed, to within 1.5–3 h following the onset of a cardiac event (Sherwood and Kristin Newby, 2014). Hs-cTnT was noted to be above the upper reference limit of 14 ng.L^−1^ at 2.8 h after the onset of symptoms, with a peak of 212 (range from 17 to 5,600) ng.L^−1^ at 12 h (Pickering et al., 2020). The bimodal release kinetics of cTn after myocardial infarction was observed by Solecki and colleagues, peak level of hs-cTnT at 12 h and a secondary peak at 82 h (Solecki et al., 2015). The kinetics of exercise-induced cTn elevation therefore might have clinical implications accordingly. The increase in hs-cTnT following exercise in the present meta-analysis was similar in pattern to that of early release kinetics observed in a clinical setting (Thygesen et al., 2018), but the peak concentration was much lower (33 ng.L^−1^) and the recovery was earlier in our meta-analysis compared with clinical conditions. As a quantitative marker of cardiomyocyte injury, the more pronounced the change of hs-cTnT, the higher the likelihood of cardiac injury (Marjot et al., 2017; Tiller et al., 2019). Concentrations lower than 50 ng L^−1^ were considered to represent micro myocardial infarction, chronic heart disease, or non-myocardial infarction conditions, such as kidney disease (Kozinski et al., 2017). The exercise-induced mean hs-cTnT elevation was therefore not indicative of pathological conditions of the heart. Although the meta-analyzed peak of cTnT occurred around 3 h post exercise, the individual studies showed peaks up to 2 h either side of the peak. Subject characteristics and exercise parameters were likely to be moderators of the time to peak, but we could not analyze such effects with our meta-analytic model. Modifying effects on the time to full recovery were also likely, as two of the three studies at the last post-exercise time point showed substantial elevations in mean hs-cTnT. As there were substantial individual variations in the peak and recovery values in our meta-analysis, further longitudinal studies are highly recommended to make the clinical implications of the findings clear.

The effects of moderators in our meta-analysis on cTnT were generally inconsistent with those in previous meta-analyses, which focused on the positive rates above the detection limit of cTn (Shave et al., 2007; Donaldson et al., 2019). Subject characteristics and fundamental exercise parameters, duration and intensity, have always been mentioned as contributors to cTn elevation previously. Donaldson and colleges found that younger age and higher heart rate was associated with increased positive rate of post-exercise cTn (including cTnT, hs-cTnT, hs-cTnI) above the detection limit (Donaldson et al., 2019). Shave et al. demonstrated that the concentration of cTn (3rd-generation assay) was higher in samples with predominantly male athletes and shorter duration endurance events, and cTnT concentrations following a cycling event was half that of running performance (27 vs. 52%) (Shave et al., 2007). In our study, the effects of moderators were analyzed from actual concentrations of pre- and post-exercise hs-cTnT, with duration and intensity included in the model to avoid any mutual confounding effects by these measures. Longer exercise duration was related to higher hs-cTnT elevations after exercise, but effects of gender and age were unclear. A difference between running and rowing was observed, but not for other exercise modalities. Although the observed trivial effect of heart rate was unclear in the current meta-analysis, the upper compatibility limit allowed for a positive effect. Exercise durations could positively influence cTn increase under similar exercise intensities (Eijsvogels et al., 2016; Gresslien and Agewall, 2016). Both mean and maximal heart rates during exercise were associated with changes in cTn in one study (Richardson et al., 2018). At similar average heart rates during exercise, hs-cTn changed more severely following intermittent exercise with higher maximal heart rates (Li et al., 2020). The duration of exercise above certain threshold heart rates therefore seemed to play an important role in hs-cTn elevation (Bjørkavoll-Bergseth et al., 2020), so more research on the effects of exercise intensity is obviously needed.

Use of the 99th percentile concentration in healthy normal reference populations was recommended as part of the decision threshold to diagnose pathological processes of the heart (Kozinski et al., 2017). There was much debate concerning the determination of the 99th percentile upper reference limit for hs-cTnT, in particular the type of population, including gender and age, should be considered (Apple et al., 2015; Mullins and Christenson, 2020). Although the effect of age was unclear in our meta-analysis, the observed effect was substantial, and we considered it prudent to estimate reference limits for different ages. The 99th percentile for hs-cTnT for male athletes at baseline and after running reduced with age, whereas in the general population the 99th percentile increased with age (Gore et al., 2014). Balmain et al. found that older age athletes (40–65 years) would have attenuated levels of hs-cTn after exercise, and the authors proposed that exercise-induced stress stimulus responses might be altered with aging (Balmain et al., 2021). Our estimates for the reference limit for the peak following exercise varied dramatically with exercise duration and to some extent with age, and have considerable uncertainty, all of which should be taken into account by clinicians using these limits as cutoff points when making medical decisions.

### Clinical Implications

It has been debated if a physiological or pathological process is responsible for the exercise-induced elevations of cTn (Eijsvogels et al., 2016). As the new assay of hs-cTn is used for quantitative assessment for cardiomyocyte damage, it is important to consider the clinical implications of related elevations in athletes. The evidence of kinetics, moderators and reference ranges in the current study has made a meaningful conclusion possible. The increase in hs-cTnT following exercise in the present meta-analysis is similar in pattern to that of early release kinetics observed in a clinical setting (Thygesen et al., 2018), but the peak concentration was much lower and the recovery occurred earlier. The exercise-induced mean hs-cTnT elevation is therefore not indicative of pathological conditions of the heart. Reference limits of baseline and hs-cTnT concentrations post-exercise for male runners should also be considered.

### Limitations

There were some limitations in the current study. More studies are needed to reduce the uncertainty in the effects of the moderators of mean hs-cTnT, especially studies of female athletes and studies of the effects of exercise after 3 days or more. The uncertainty in the effects would likely have been reduced if authors had all used log-transformation in their analyses: our conversion of raw mean and median effects to log-transformed means will have reduced bias, but possibly at the expense of additional uncertainty. The magnitudes of the random-effect SDs were unclear, but the observed substantial effects represent differences between study-estimates that were not explained by the meta-analytic model, either because of non-linear effects and interactions of the numeric moderators, substantial moderators not included in the model (e.g., the health status of athletes and recent and long-term exercise history et al.), and setting-specific factors (e.g., assay technique and data-analysis methods). More studies would reduce the uncertainty in these differences and might also reduce their magnitude by allowing development of a more comprehensive model. The meta-analysis of SDs, representing individual variations at rest and in response to exercise, would require an impractically larger amount of data for adequate precision of the estimates. Successful meta-analysis of factors responsible for individual differences would be possible, if enough authors analyzed and presented the modifying effects of subject characteristics; the effect of each characteristic could then be the dependent variable in separate meta-analyses.

## Conclusion

This meta-analysis has established the kinetics of cTnT elevation after exercise in athletic populations and has identified exercise duration as a moderator of the elevations. Meta-analyses of the means and standard deviations before and after exercise have provided upper reference limits for male runners.

## Data Availability Statement

The original contributions presented in the study are included in the article/supplementary material further inquiries can be directed to the corresponding author/s.

## Author Contributions

FL, XW, JN, and LY conceived and designed the study. FL, WH, KW, and LY selected the papers and extracted the data. WH and FL conducted the data analysis. FL, WH, XW, and JN interpreted the data. FL and WH wrote the draft. FL, WH, XW, JB, JN, JQ, BQ, and LY wrote the final version. All authors agree with the results and conclusions of this study.

## Conflict of Interest

The authors declare that the research was conducted in the absence of any commercial or financial relationships that could be construed as a potential conflict of interest.
